# A Vision-Based Machine Learning Method for Barrier Access Control Using Vehicle License Plate Authentication

**DOI:** 10.3390/s20123578

**Published:** 2020-06-24

**Authors:** Kh Tohidul Islam, Ram Gopal Raj, Syed Mohammed Shamsul Islam, Sudanthi Wijewickrema, Md Sazzad Hossain, Tayla Razmovski, Stephen O’Leary

**Affiliations:** 1 Department of Artificial Intelligence, Faculty of Computer Science & Information Technology, University of Malaya, Kuala Lumpur 50603, Malaysia; kh.tohidulislam@gmail.com; 2 Department of Surgery (Otolaryngology), Faculty of Medicine, Dentistry and Health Sciences, University of Melbourne, Melbourne, VIC 3010, Australia; swijewickrem@unimelb.edu.au (S.W.); tayla.razmovski@gmail.com (T.R.); sjoleary@unimelb.edu.au (S.O.); 3 Discipline of Computing and Security, School of Science, Edith Cowan University (ECU), Joondalup, WA 6027, Australia; syed.islam@ecu.edu.au; 4 Department of Computer Science and Software Engineering, The University of Western Australia, Crawley, WA 6009, Australia; 5 Faculty of Information Technology, Monash University, Melbourne, VIC 3800, Australia; sazzad.hossain@monash.edu

**Keywords:** automatic license plate recognition, intelligent vehicle access, histogram of oriented gradients, artificial neural networks

## Abstract

Automatic vehicle license plate recognition is an essential part of intelligent vehicle access control and monitoring systems. With the increasing number of vehicles, it is important that an effective real-time system for automated license plate recognition is developed. Computer vision techniques are typically used for this task. However, it remains a challenging problem, as both high accuracy and low processing time are required in such a system. Here, we propose a method for license plate recognition that seeks to find a balance between these two requirements. The proposed method consists of two stages: detection and recognition. In the detection stage, the image is processed so that a region of interest is identified. In the recognition stage, features are extracted from the region of interest using the histogram of oriented gradients method. These features are then used to train an artificial neural network to identify characters in the license plate. Experimental results show that the proposed method achieves a high level of accuracy as well as low processing time when compared to existing methods, indicating that it is suitable for real-time applications.

## 1. Introduction

Automatic vehicle license plate recognition (AVLPR) is used in a wide range of applications including automatic vehicle access control, traffic monitoring, and automatic toll and parking payment systems. Implementation of AVLPR systems is challenging due to the complexity of the natural images from which the license plates need to be extracted, and the real-time nature of the application. An AVLPR system depends on the quality of its physical components which acquire images and the algorithms that process the acquired images. In this paper, we focus on the algorithmic aspects of an AVLPR system, which includes the localization of a vehicle license plate, character extraction and character recognition. For the process of license plate localization, researchers have proposed various methods including, connected component analysis (CCA) [1], morphological analysis with edge statistics [2], edge point analysis [3], color processing [4], and deep learning [5]. The rate of accuracy for these localization methods varies from 80.00% to 99.80% [3,6,7]. The methods most commonly used for the recognition stage are optical character recognition (OCR) [8,9], template matching [10,11], feature extraction and classification [12,13], and deep learning based methods [5,14].

In recent years, several countries including the United Kingdom, United States of America, Australia, China, and Canada have successfully used real-time AVLPR in intelligent transport systems [3,15,16]. However, this is not yet widely used in Malaysia. The road transportation department of Malaysia has authorized the use of three types of license plates. The first contains white alphanumeric characters embossed or pasted on a black background. The second type is allocated to vehicles belonging to diplomatic personnel and also contains white alphanumeric characters, but the background on these plates is red. The third type is assigned to taxi cabs and hired vehicles, consisting of black alphanumeric characters on a white plate. There are also some rules the characters must satisfy, for example, there are no leading zeros in a license plate sequence. Additionally, the letters “I” and “O” are excluded from the sequences due to their similarities with the numbers “1” and “0” and only military vehicles have the letter “Z” included in their license plates. The objective of this study was to implement a fast and accurate method for automatic recognition of Malaysian license plates. Additionally, this method can be easily applied to similar datasets.

The paper is organized as follows. A discussion of the existing literature in the field is given in Section 2. In Section 3, we introduce the proposed method: localizing the license plate based on a deep learning method for object detection, image feature extraction through histogram of oriented gradients (HOG), and character recognition using an artificial neural network (ANN) [17]. In Section 4, we establish the suitability of the method for real-time license plate detection through experiments, including comparisons with similar methods. The paper concludes in Section 5 with a discussion of the findings.

## 2. Related Work

A typical AVLPR system consists of three stages: detection or localization of the region of interest (i.e., the license plate from the image), character extraction from the license plate, and recognition of those characters [18,19,20].

### 2.1. Detection or Localization

The precision of an AVLPR system is typically influenced by the license plate detection stage. As such, many researchers have focused on license detection as a priority. For instance, Suryanarayana et al. [21] and Mahini et al. [22] used the Sobel gradient operator, CCA, and morphological operations to extract the license plate region. They reported 95.00% and 96.5% correct localization, respectively. To monitor highway ticketing systems, a hybrid edge-detection-based method for segmenting the vehicle license plate region was introduced by Hongliang and Changping [2], achieving 99.60% detection accuracy. According to Zheng et al. [23], if the vertical edges of the vehicle image are extracted while the edges representing the background and noise are removed, the vehicle license plate can be easily segmented from the resultant image. In their findings, the overall segmentation accuracy reported was approximately 97%. Luo et al. [24] proposed a license plate detection system for Chinese vehicles, where a single-shot multi-box detector was used for the detection method [25] and achieved 96.5% detection accuracy on their database.

A wavelet transform based method was applied by Hsieh et al. [26] to detect the license plate region from a complex background. They successfully localized the vehicle license plate in three steps. Firstly, they used Haar scaling [27] as a function for wavelet transformation. Secondly, they roughly localized the vehicle license plate by finding the reference line with the maximum horizontal variation in the transformed image. Finally, they localized the license plate region below the reference line by calculating the total pixel values (the region with the maximum pixel value was considered as the plate region) followed by geometric verification using metrics such as the ratio of length and width of the region. They achieved 92.40% detection accuracy on average. A feature-based hybrid method for vehicle license plate detection was introduced by Niu et al. [28]. Initially, they used color processing (blue–white pairs) for possible localization of the license plate. They then used morphological processing such as open and close operations, followed by CCA. They used geometrical features (e.g., size) to remove unnecessary small regions and finally used HOG features in a support vector machine (SVM) to detect the vehicle license plate and achieved 98.06% detection accuracy with their database.

### 2.2. License Plate Character Segmentation

Correct segmentation of license plate characters is important, as the majority of incorrect recognition is due to incorrect segmentation, as opposed to issues in the recognition process [29]. Several methods have been introduced for character segmentation. For instance, Arafat et al. [9] proposed a license plate character segmentation method based on CCA. The detected license plate region was converted into a binary image and eight connected components were used for character region labeling. They achieved 95.40% character segmentation accuracy. A similar method was also introduced by Tabrizi et al. [13], where they achieved 95.24% character segmentation accuracy.

Chai and Zuo [30] used a similar process for segmenting vehicle license plate characters. To remove unnecessary small character regions from the detected license plate, a vertical and horizontal projection method, alongside morphological operations and CCA, was used. They achieved 97.00% character segmentation accuracy. Dhar et al. [31] proposed a vehicle license plate recognition system for Bangladeshi vehicles using edge detection and deep learning. Their method for character segmentation involved a combination of edge detection, morphological operations, and analysis of segmented region properties (e.g., ratio of height and width). Although they did not mention any segmentation results, they achieved 99.6% accuracy for license plate recognition. De Gaetano Ariel et al. [32] introduced an algorithm for Argentinian license plate character segmentation. They used horizontal and vertical edge projection to extract the characters, with a 96.49% accuracy level.

### 2.3. Recognition or Classification

Some researchers recognized license plates using adaptive boosting in conjunction with Haar-like features and training cascade classifiers on those features [33,34,35]. Several researchers have used template matching to recognize the license plate text [10,11]. Feature extraction based recognition has also proven to be accurate in vehicle license plate recognition [12,13,28]. Samma et al. [12] introduced fuzzy support vector machines (FSVM) with particle swarm optimization for Malaysian vehicle license plate recognition. They extracted image features using Haar-like wavelet functions and using a FSVM for classification and they achieved 98.36% recognition accuracy. A hybrid k-nearest neighbors and support vector machine (KNN-SVM) based vehicle license plate recognition system was proposed by Tabrizi et al. [13]. They used operations such as filling, filtering, dilation, and edge detection (using the Prewitt operator [36]) for license plate localization after color to grayscale conversion. For feature extraction, they used a structural and zoning feature extraction method. Initially, a KNN was trained with all possible classes including similar and dissimilar characters (whereas the SVM was trained only on similar character samples). Once the KNN ascertained which “similar character” class the target character belonged to, the SVM performed the next stage of classification to determine the actual class. They achieved 97.03% recognition accuracy.

Thakur et al. [37] introduced an approach that used a genetic algorithm (GA) for feature extraction and a neural network (NN) for classification in order to identify characters in vehicle license plates. They achieved 97.00% classification accuracy. Jin et al. [3] introduced a solution for license plate recognition in China. They used hand-crafted features on a fuzzy classifier to obtain 92.00% recognition accuracy. Another group of researchers proposed a radial wavelet neural network for vehicle license plate recognition [38]. They achieved 99.54% recognition accuracy.

Brillantes et al. [39] utilized fuzzy logic for Filipino vehicle license plate recognition. Their method was effective in identifying license plates from different issues which contained characters of different fonts and styles. They segmented the characters using CCA along with fuzzy clustering. They then used a template matching algorithm to recognize the segmented characters. The recognition accuracy of their methods was 95.00%. Another fuzzy based license plate region segmentation method was introduced by Mukherjee et al. [40]. They used fuzzy logic to identify edges in the license plate in conjunction with other edge detection algorithms such as Canny and Sobel [41,42]. A template matching algorithm was then used to recognize the license plate text from the segmented region and achieved a recognition accuracy of 79.30%. A hybrid segmentation method combining fuzzy logic and k-means clustering was proposed by Olmí et al. [43] for vehicle license plate region extraction. They developed SVM and ANN models to perform the classification task and achieved an accuracy level of 95.30%.

### 2.4. Recent Methods of AVLPR

Recently, deep learning based image classification approaches have received more attention from researchers as they can learn image features on their own, in addition to performing classification [44]. Therefore, no feature extraction is required for deep learning approaches. However, despite the advantages of using deep learning in image classification, it requires a large training image database and very high computational power. Li et al. [45] investigated a method of identifying similar characters on license plates based on convolution neural networks (CNN). They used CNNs as feature extractors and also as classifiers. They achieved 97.20% classification accuracy. Another deep learning method based on the AlexNet [46] was introduced by Lee et al. [47] for AVLPR, where they re-trained the AlexNet to perform their task on their database and achieved 95.24% correct recognition. Rizvi et al. [5] also proposed a deep learning based approach for Italian vehicle license plate recognition on a mobile platform. They utilized two deep learning models, one to detect and localize the license plate and the characters present, and another as a character classifier. They achieved 98.00% recognition accuracy with their database.

Another deep learning method called “you only look once” (YOLO) was developed for real-time object detection, which is now being used in AVLPR [48]. For example, Kessentini et al. [49] proposed a two-stage deep learning approach that first used YOLO version 2 (YOLO v2) for license plate detection [50]. Then, they used a convolutional recurrent neural network (CRNN) based segmentation-free approach for license plate character recognition. They achieved 95.31% and 99.49% character recognition accuracy in the two stages, respectively. Another YOLO based method was developed by Hendry and Chen [51] for vehicle license plate recognition in Taiwan. Here, for each character, detection and recognition was carried out using a YOLO model, totaling 36 YOLO models used for 36 classes. They achieved 98.22% and 78.00% accuracy for vehicle license plate detection and recognition, respectively.

Similarly, Yonetsu et al. [52] also introduced a two-stage YOLO v2 model for Japanese license plate detection. To increase accuracy, they initially detected the vehicle, followed by the detection of the license plate. In clear weather conditions, they achieved 99.00% and 87.00% accuracy for vehicle and license plate detection, respectively. A YOLO based three-stage Bangladeshi vehicle license plate detection and recognition method was implemented by Abdullah et al. [53]. Firstly, they used YOLO version 3 (YOLOv3) as their detection model [54]. In the second stage, they segmented the license plate region and character patches. Finally, they used a ResNet-20 deep learning model for the character recognition [55]. They achieved 95.00% and 92.70% accuracy for license plate detection character recognition, respectively. Laroca et al. [56] used YOLO for license plate detection and then another method proposed by Silva and Jung [57] for character segmentation and recognition. They tested their performance on their own database (UFPR-ALPR), which is now publicly available for research purposes. They achieved 98.33% and 93.53% accuracy for vehicle license plate detection and recognition, respectively.

## 3. Methodology

**Figure 1:**
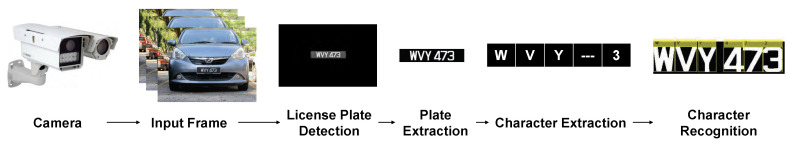
Outline of the proposed system for automatic license plate recognition.

In the proposed system, a digital camera was placed at a fixed distance and height to be able to capture images of vehicle license plates. When a vehicle is at a predefined distance from the camera, it captures an image of the front of the vehicle, including the license plate. This image then went through several pre-processing steps to eliminate the unwanted background and localize the license plate region. Once this region was extracted from the original image, a character segmentation algorithm was used to segment the characters from the background of the license plate. The segmented characters were then identified using an ANN classifier trained on HOG features. Figure 1 illustrates the steps involved for the proposed AVLPR system.

### 3.1. Image Acquisition

A digital camera was used as an image acquisition device. This camera was placed at a height of 0.5 m from the ground. An ideal distance to capture images of an arriving vehicle was pre-defined. To detect whether a vehicle was within this pre-defined distance threshold, we subtracted the image of the background (with no vehicles) from each frame of the obtained video. If more than 70% of the background was obscured, it was considered that a vehicle was within this threshold. To avoid unnecessary background information, the camera lens was set to 5× zoom. The speed of the vehicles when the images were captured was around 20 km/h. Camera specifications and image acquisition properties are shown in Table 1.

### 3.2. Detection of the License Plate

**Figure 2:**
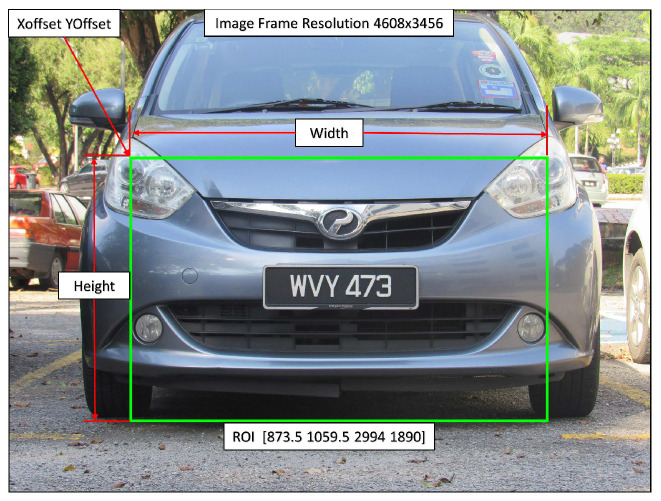
The initial extracted region of interest, defined by the green bounding box.

Once the image was acquired, it was then processed to detect the license plate. First, the contrast of the RGB (red, green, and blue) images was improved using histogram equalization. As the location of the license plate in the acquired images was relatively consistent, we extracted a pre-defined rectangular region from the image to be used in the next stages of processing. Figure 2 shows the specifications of the region of interest (ROI). Therefore, we reduced the size of the image to be processed from 4608×3456 pixels in the original image to 2995×1891 pixels. The region to be extracted was defined using the x and y coordinates of the upper left corner (XOffset and YOffset) and the width and height of the rectangle.

**Figure 3:**
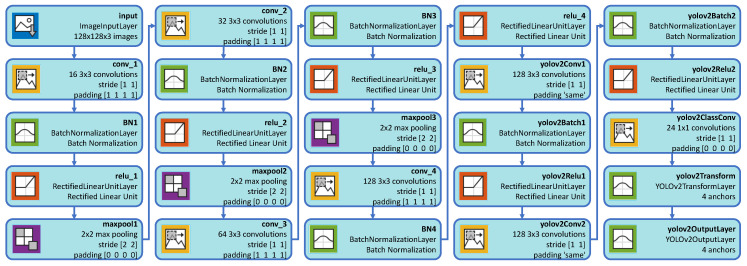
The architecture of YOLO V2 network.

To detect the license plate, the ROI extracted in the previous stage was first resized to 128×128 pixels. Then, based on previous studies, a deep learning based approach (YOLO v2, as discussed in [50]) was used to extract the license plate region. The network accepts 128×128 pixels RGB images as an input and processes them in an end-to-end manner to produce a corresponding bounding box for the license plate region. This network has 25 layers: one Image Input layer, seven Convolution layers, six Batch Normalization layers, six Rectified Linear Unit (ReLU) layers, three Max Pooling layers, one YOLO v2 Transform Layer, and one YOLO v2 Output layer [58]. Figure 3 shows the YOLO v2 network architecture. We used this network to detect the license plate region only, not for license plate character recognition. The motivation behind this methodology is to reduce total processing time with low computational power without compromising the accuracy.

**Figure 4:**
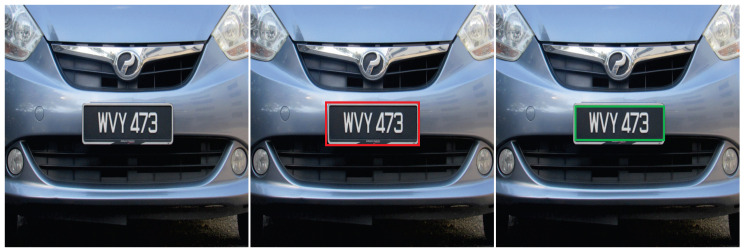
Detection result: from left to right, input image, ground truth image (red bounding box), and output image with detected license plate ROI (green bounding box).

For training, we used the stochastic gradient descent with momentum (SGDM) optimizer of 0.9, Initial Learn Rate of 0.001, and Max Epochs of 30 [59]. We chose these values as they provided the best performance with low computational power in our experiments. To improve the network accuracy, we used training image augmentation by randomly flipping the images during the training phase. By using this image augmentation, we increased the variations in the training images without actually having to increase the number of labeled images. Note that, to observe unbiased evaluation, we did not perform any augmentation to test images and preserved it as unmodified. An example detection result is shown in Figure 4.

### 3.3. Alphanumeric Character Segmentation

**Figure 5:**
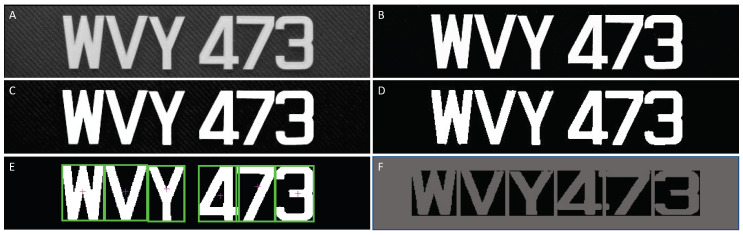
Alphanumeric character segmentation: (**A**) before gray level histogram equalization; (**B**) after gray level histogram equalization; (**C**) median filtered and noise removed image; (**D**) binary image; (**E**) character region segmentation; and (**F**) extracted and resized characters.

In the next stage, the alphanumeric characters that made up the license plate were extracted from the region resulting from the previous step. To this end, we first applied a gray level histogram equalization algorithm to increase the contrast level of the license plate region. Next, we converted the resulting image into a binary image using a global threshold of 0.5 (in the range [0, 1]). Then, the image was smoothed using a 3×3 median filter and salt-and-paper noise was removed from it. Any object that remained in the binary image after these operations was considered to represent a character. We segmented these characters using CCA. Each segmented character was resized to 56×56 pixels. The character segmentation process is illustrated in Figure 5 and Algorithm 1.
**Algorithm 1:** Alphanumeric character segmentation algorithm.
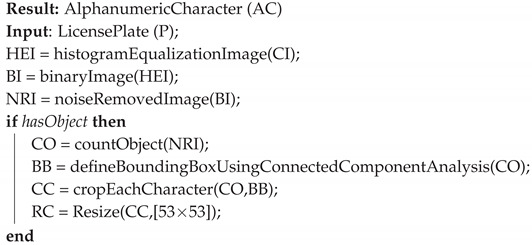


### 3.4. Feature Extraction

**Figure 6:**
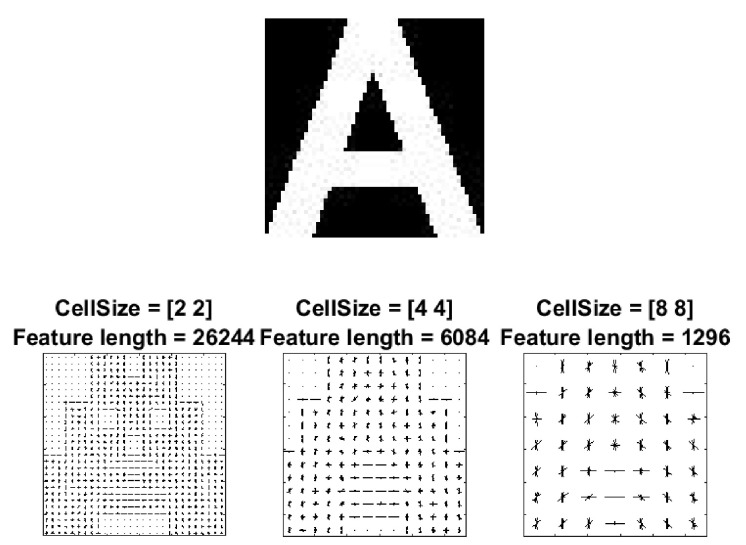
HOG feature vector visualization with different cell sizes.

To be able to identify the characters of a license plate, we needed to first extract features from them that define their characteristics. For this purpose, we used HOG features [17] as it has successfully been used in many applications. To calculate the HoG features, the image was subdivided into smaller neighborhood regions (or “cells”) [60]. Then, for each cell, at each pixel, the kernels [−1,0,+1] and ${\left[-1,0,+1\right]}^{T}$ were applied to get the horizontal ($G_{x}$) and vertical ($G_{y}$) edge values, respectively. The magnitude and orientation of the gradient were calculated as $M\left(x,y\right)=\sqrt{\left(G_{x}^{2}+G_{y}^{2}\right)}$ and $\theta \left(x,y\right)=tan^{-1}\left(\frac{G_{y}}{G_{x}}\right)$, respectively. Histograms of the unsigned angle (0°to 180°) weighted on the magnitude of each cell were then generated. Cells were combined into blocks and block normalization was performed on the concatenated histograms to account for variations in illumination. The length of the resulting feature vector depends on factors such as image size, cell size, and bin width of the histograms. For the proposed method, we used a cell size of 4×4 and each histogram was comprised of 9 bins, in order to achieve a balance between accuracy and efficiency. The resulting feature vector was of size 1×6084. Figure 6 gives a visualization of HOG features for different cell sizes.

### 3.5. Artificial Neural Network (ANN) Architecture

**Figure 7:**
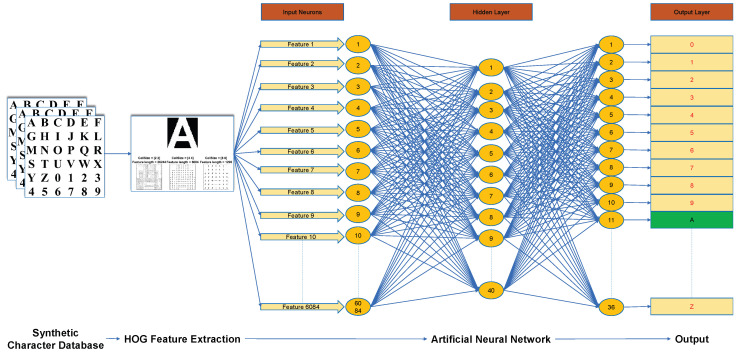
Proposed recognition process along with the artificial neural network architecture.

Once the features were extracted from the segmented characters, we trained an artificial neural network to identify them. As each character was encoded using a 1×6084 feature vector, the ANN had 6084 input neurons. The hidden layer comprised 40 neurons and the output layer had 36, equal to the number of different alphanumeric characters under consideration. Figure 7 shows the proposed recognition process along with the architecture of the ANN.

## 4. Experimental Results

Initially, we created a synthetic character image database to train and test the classification method. In addition, we created a database of real images, using the image acquisition method discussed above, to test the proposed method. We justified the selection of HOG as the feature extraction method and ANN as the classifier by comparing its performance with other feature extraction and classification methods. We also compared the performance of our method to similar existing methods. To conduct these experiments, an Intel^®^Core^™^i5 computer with 8 Gigabytes of RAM, running Windows Professional 64-bit operating system was used. MATLAB^®^ framework (MathWorks, Natick, MA, USA) was used for the implementation of the proposed method as well as the experiments.

### 4.1. Generation of a Synthetic Data for Training

**Figure 8:**
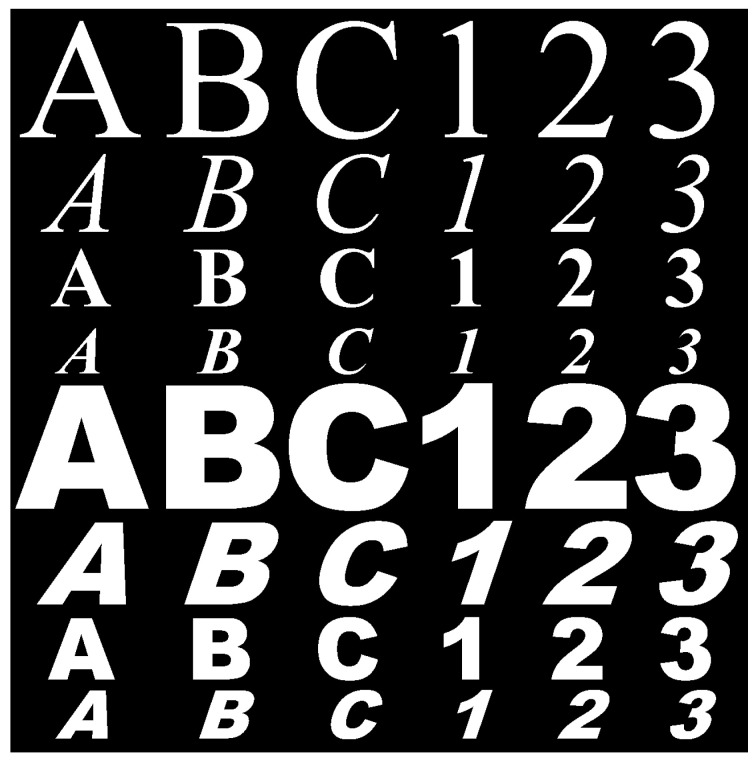
Synthetic samples for training purposes.

Using synthetic images is convenient as it enables the creation of a variety of training samples without having to manually collect them. The database was created by generating characters using random fonts and sizes (12—72 in steps of 2). In addition, one of four styles (normal, bold, italic, or bold with italic) was also used in the font generation process. To align with the type of characters available in license plates, we included the 10 numerals (0–9) as well as the characters of the English alphabet (A–Z). We also rotated the characters in the database by a randomly chosen angle between ±0° and ±15°. Each class contained 200 samples consisting of an equal number of rotated and unrotated images. In total, 36×200=7200 images were used to train the ANN. Some characters from the synthetic training database are shown in Figure 8.

### 4.2. Performance on Synthetic Data

The ANN, discussed in Section 3, was trained on 70% of the synthetic database (5040 randomly selected samples). The remaining images were split evenly for validation and testing (1080 samples each). To determine the ideal number of hidden neurons, ANNs with different numbers of neurons (10, 20, and 40) were trained on these data five times each (see Table 2). The network with the best performance (with 40 hidden neurons) was selected as our trained network. We further increased the number of neurons to 60 and recorded comparably high processing time without reducing the error (%). The overall accuracy of the classification was 99.90% and the only misclassifications were between the classes **0** and **O**.

### 4.3. Performance on Real Data

**Figure 9:**
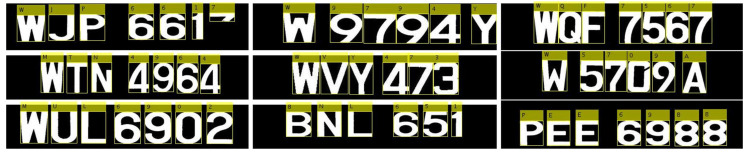
Real-time experimental results for license plate character recognition.

To test the performance on real-data, we acquired images at several locations in Kuala Lumpur, Malaysia using the process discussed in Section 3.1. In total, 100 vehicle license plates were used in this experiment, where each plate contained 5–8 alphanumeric characters. Then, 671 characters were extracted from the license plate images using the process discussed above. Classes **I**, **O**, and **Z** were not used here as they are not present in the license plates, as discussed above. The number of characters in each class is shown in Table 3. An accuracy level of 99.70% was achieved. The misclassifications in this experiment occurred among the classes **S**, **5**, and **9**, likely due to similarities in the characters. Real-time classification results for a sample license plate image are shown in Figure 9.

### 4.4. Comparison of Different Feature Extraction and Classification Methods

We also compared the proposed method with combinations of feature extraction and classification methods with respect to accuracy and processing time. Bag of words (BoF) [61], scale-invariant feature transform (SIFT) [62], and HOG were the feature extraction methods used. The classifiers used were: stacked auto-encoders (SAE), k-nearest neighbors (KNN), support vector machines (SVM), and ANN [63]. Processing time was calculated as the average time taken for the procedure to complete, as discussed in Section 3 (license plate extraction, characters extraction, feature extraction, and classification). The same 100 images used in the previous experiment were used here. The results are shown in Table 4.

### 4.5. Performance Comparison with Other Similar Methods

Table 5 compares the performance of the proposed algorithm with other similar AVLPR methods in the literature. We reimplemented these methods on our system and trained and tested them on the same datasets to ensure an unbiased comparison. The training and testing was performed on our synthetic and real image databases, respectively. Note that the processing time only reports the time taken for the feature extraction and classification stages of the process. Accuracy denotes the classification accuracy. Since the training dataset was balanced, we did not consider bias performance metrics such as sensitivity and precision. As can be seen in Table 5, the proposed method outperformed the other compared methods.

### 4.6. Comparison of Methods with Respect to the Medialab Database

We compared the performance of our method to the methods discussed above, on a publicly available database (Medialab LPR (License Plate Recognition) database [64]) to investigate the transferability of results. This database contains still images and video sequences captured at various times of the day, under different weather conditions. We included all still images from this database except for ones that contained more than one vehicle. As our image capture system was specifically designed to capture only one vehicle at a time in order to simplify the subsequent image processing steps, images with multiple vehicles are beyond our scope.

The methods were compared with respect to the different stages of a typical AVLPR system (detection, character segmentation, and classification). Table 6 shows the comparison results (detection, character segmentation, and classification accuracy show the percentages of vehicle number plate regions detected, characters accurately extracted, and accurate classifications, respectively). As our method of pre-defined rectangular region selection (shown in Figure 2) was specifically designed for our image acquisition system, we did not consider this step in the comparison as different image acquisition systems were used in obtaining images for this database. Instead, the full image was used for detecting the license plate.

Note that some of the methods in Table 6 do not address all three stages of the process. This is due to the fact that some papers only performed the latter parts of the process (for example, using pre-segmented characters for classification) and others did not clearly mention the methods they used. All methods were trained on our synthetic database as discussed above (no retraining was performed on the Medialab LPR database).

As can be seen in Table 6, the proposed method outperformed the other methods compared. However, the overall classification performance for all methods was slightly lower than that on our database (Table 5). We hypothesize that it could be due to factors such as differences in resolution and capture conditions (for example, weather, and time of day) of the images in the two databases.

### 4.7. Comparison of Methods with Respect to the UFPR-ALPR Database

We further compared the performance of our method with other existing methods (discussed above) on another publicly available database (UFPR-ALPR), which includes images that are more challenging [56]. This database contains multiple images of 150 vehicles (including motorcycles and cars) from real-world scenarios. The images were collected from different devices such as mobile cameras (iPhone^®^ 7 Plus and Huawei^®^ P9 Lite) and GoPro^®^ HERO^®^4 Silver.

Since we used a digital camera to capture images in our method, we only considered those images in the UFPR-ALPR database that were captured by a similar device (GoPro^®^ HERO^®^4 Silver). The database is split into three sets: 40%, 40%, and 20% for training, testing, and validation, respectively. We only considered the testing portion of the database to perform this comparison. In addition, we did not consider motorcycle images here, as our method was developed for cars. First, we converted the original image from 1920×1080 to 1498×946 pixels (to keep it consistent with the images of our database). Then, we performed the detection and recognition processes on the resized images.

As can be seen in Table 7, the proposed method outperformed the other methods with respect to accuracy of detection, segmentation, and classification. However, the overall classification performance for all methods were lower than on ours and Medialab LPR databases (Table 5 and Table 6). We hypothesize that this could be due to factors such as the uncontrolled capture conditions (i.e, speed of vehicle, weather, and time of day) of the images in the three databases.

## 5. Conclusions

In this paper, we propose a methodology for automatic vehicle license plate detection and recognition. This process consists of the following steps: image acquisition, license plate extraction, character extraction, and recognition. We demonstrated through experiments on synthetic and real license plate data that the proposed system is not only highly accurate but is also efficient. We also compared this method to similar existing methods and showed that it achieved a balance between accuracy and efficiency, and as such is suitable for real-time detection of license plates. A limitation of this work is that our method was only tested on a database of Malaysian license plate images captured by the researchers and the publicly available Medialab LPR and UFPR-ALPR databases. In the future, we will explore how it performs on other license plate databases. We will also investigate the use of multi-stage deep learning architectures (detection and recognition) in this domain. Furthermore, since we proposed barrier access control for the controlled environment, the current image acquisition system was set up so that only one vehicle was visible in the field of view. As a result, the image only captured one vehicle per frame, simplifying the process of license plate detection. In future work, we will extend our methodology to identify license plates using more complex images containing multiple vehicles. In addition, we will increase the size of the training database in an effort to minimise misclassification of similar classes.

## Figures and Tables

**Table 1:** Image acquisition properties.

Name	Description
Image acquisition device name	Canon^®^ Power Shot SX530 HS
Zooming capabilities	50× Optical zoom
Camera zooming position	5× Optical zoom
Weather	Daylight, rainy, sunny, cloudy
Capturing period	Day and Night
Background	Complex; not fixed
Horizontal field-of-view	Approximately 75°
Image dimension	4608×3456
Vehicle speed limit	20 km/h; 5.56 m/s
Capturing distance	15 meter

**Table 2:** Training performance with respect to hidden neuron size. The best performance is highlighted in bold.

Hidden Neurons	Repetition Number	Iterations	Time	Performance	Gradient	Error (%)
10	1	160	0:00:45	1.47 × 10${}^{-3}$	9.03 × 10${}^{-4}$	1.50 × 10${}^{-0}$
	2	179	0:00:51	8.10 × 10${}^{-4}$	9.09 × 10${}^{-3}$	6.11 × 10${}^{-1}$
	3	273	0:01:17	6.00 × 10${}^{-4}$	9.62 × 10${}^{-4}$	8.06 × 10${}^{-1}$
	4	189	0:00:53	2.02 × 10${}^{-3}$	1.34 × 10${}^{-3}$	1.86 × 10${}^{-0}$
	5	214	0:01:00	1.34 × 10${}^{-3}$	6.66 × 10${}^{-4}$	1.19 × 10${}^{-0}$
20	1	167	0:01:07	2.00 × 10${}^{-4}$	1.04 × 10${}^{-3}$	2.50 × 10${}^{-1}$
	2	186	0:01:18	4.86 × 10${}^{-6}$	1.97 × 10${}^{-4}$	2.50 × 10${}^{-1}$
	3	140	0:00:59	1.51 × 10${}^{-4}$	6.95 × 10${}^{-4}$	3.33 × 10${}^{-1}$
	4	165	0:01:10	6.12 × 10${}^{-5}$	2.72 × 10${}^{-4}$	2.22 × 10${}^{-1}$
	5	137	0:00:58	1.04 × 10${}^{-4}$	5.59 × 10${}^{-4}$	3.61 × 10${}^{-1}$
**40**	1	124	0:01:26	3.65 × 10${}^{-5}$	2.35 × 10${}^{-4}$	2.78 × 10${}^{-1}$
	2	124	0:01:25	2.78 × 10${}^{-5}$	2.12 × 10${}^{-4}$	1.67 × 10${}^{-1}$
	**3**	**151**	**0:01:44**	**1.29 × 10${}^{-5}$**	**7.51 × 10${}^{-4}$**	**1.11 × 10${}^{-1}$**
	4	142	0:02:03	7.50 × 10${}^{-6}$	3.67 × 10${}^{-4}$	1.67 × 10${}^{-1}$
	5	105	0:01:18	1.22 × 10${}^{-4}$	1.81 × 10${}^{-3}$	3.33 × 10${}^{-1}$

**Table 3:** The number of characters extracted from license plate images in each class.

Characters	0	1	2	3	4	5	6	7	8	9	A
Quantity	29	37	36	40	40	41	39	51	35	33	13
**Characters**	**B**	**C**	**D**	**E**	**F**	**G**	**H**	**J**	**K**	**L**	**M**
Quantity	16	7	7	10	5	7	7	13	12	12	7
**Characters**	**N**	**P**	**Q**	**R**	**S**	**T**	**U**	**V**	**W**	**X**	**Y**
Quantity	10	15	9	12	9	13	3	15	73	6	9

**Table 4:** Comparison of performance for different combinations of feature extraction and classification methods. The best performance per metric is highlighted in bold.

Method	Processing Time (s)					Accuracy (%)
	Plate Extraction	Character Extraction	Feature Extraction	Classification	Total	
BoF+SAE	0.15	0.25	0.21	0.015	0.625	95.73
BoF+KNN	0.15	0.25	0.21	0.024	0.634	88.25
BoF+SVM	0.15	0.25	0.21	0.020	0.630	89.78
BoF+ANN	0.15	0.25	0.21	0.021	0.631	98.33
SIFT+SAE	0.15	0.25	0.28	0.019	0.699	93.75
SIFT+KNN	0.15	0.25	0.28	0.030	0.710	87.38
SIFT+SVM	0.15	0.25	0.28	0.027	0.707	88.94
SIFT+ANN	0.15	0.25	0.28	0.026	0.706	96.18
HOG+SAE	0.15	0.25	0.27	0.018	0.688	94.30
HOG+KNN	0.15	0.25	0.27	0.028	0.698	97.60
HOG+SVM	0.15	0.25	0.27	0.025	0.695	98.90
**Proposed (HOG+ANN)**	**0.15**	**0.25**	**0.27**	**0.010**	**0.690**	**99.70**

**Table 5:** The classification performance for the proposed method compared to similar existing methods. The best performance per metric is highlighted in bold.

Method	Feature Extraction Method	Classifier	Total Time (s)	Accuracy (%)
Jin et al. [3]	Hand-Crafted	Fuzzy	0.432	92.00
Arafat et al. [9]	OCR	OCR	0.681	97.86
Samma et al. [12]	Haar-like	FSVM	0.649	98.36
Tabrizi et al. [13]	KNN+SVM	KNN+SVM	0.721	97.03
Niu et al. [28]	HOG	SVM	0.645	96.60
Li et al. [45]	CNN	CNN	0.825	99.20
Thakur et al. [37]	GA	ANN	0.532	97.00
Cheng et al. [38]	SCDCS-LS	RWNN	0.659	99.54
Lee et al. [47]	AlexNet	AlexNet	0.983	99.58
**Proposed**	**HOG**	**ANN**	**0.280**	**99.70**

**Table 6:** Performance comparison on the Medialab LPR database. The stages that were not addressed in the original papers are denoted by “—”. The best performance per metric is highlighted in bold.

Method	Accuracy (%)		
	Detection	Segmentation	Classification
Jin et al. [3]	95.73	98.87	91.25
Arafat et al. [9]	98.30	99.30	96.57
Samma et al. [12]	96.25	—	98.05
Tabrizi et al. [13]	96.98	96.85	96.54
Niu et al. [28]	98.45	—	96.38
Li et al. [45]	—	—	98.52
Thakur et al. [37]	97.85	98.37	97.35
Cheng et al. [38]	—	—	99.38
Lee et al. [47]	—	—	97.38
**Proposed**	**99.30**	**99.45**	**99.50**

**Table 7:** Performance comparison on the UFPR-ALPR database. The stages that were not addressed in the original papers are denoted by “—”. The best performance per metric is highlighted in bold.

Method	Accuracy (%)		
	Detection	Segmentation	Classification
Jin et al. [3]	85.48	91.75	85.35
Arafat et al. [9]	85.45	93.45	90.37
Samma et al. [12]	80.35	—	91.70
Tabrizi et al. [13]	84.45	90.50	92.86
Niu et al. [28]	85.80	—	89.32
Li et al. [45]	—	—	92.71
Thakur et al. [37]	82.35	91.22	90.85
Cheng et al. [38]	—	—	92.50
Lee et al. [47]	—	—	92.75
**Proposed**	**98.45**	**93.85**	**95.80**

## Data Availability

The datasets used in this study are available from the corresponding author upon request.

## Abbreviations

The following abbreviations are used in this manuscript:

ANN	artificial Neural Network
AVLPR	automatic vehicle license plate recognition
BoF	bag of words
CCA	connected component analysis
CNN	convolution neural network
FSVM	fuzzy support vector machines
GA	genetic algorithm
HOG	histogram of oriented gradients
KNN	k-nearest neighbors
NN	neural network
OCR	optical character recognition
ReLU	rectified linear unit
RGB	red, green, and blue
ROC	receiver operating characteristic
ROI	region of interest
SAE	stacked auto-encoders
SIFT	scale-invariant feature transform
SGDM	stochastic gradient descent with momentum
SVM	support Vector Machine
YOLO	you only look once
